# Evaluation of ethnic disparities in detection of depression and anxiety in primary care during the maternal period: combined analysis of routine and cohort data

**DOI:** 10.1192/bjp.bp.114.158832

**Published:** 2016-05

**Authors:** Stephanie L. Prady, Kate E. Pickett, Emily S. Petherick, Simon Gilbody, Tim Croudace, Dan Mason, Trevor A. Sheldon, John Wright

**Affiliations:** **Stephanie L. Prady**, PhD, **Kate E. Pickett**, PhD, Department of Health Sciences, University of York, York; **Emily S. Petherick**, PhD, Bradford Institute for Health Research, Bradford Royal Infirmary, Bradford; **Simon Gilbody**, DPhil, FRCPsych, **Tim Croudace**, PhD, Department of Health Sciences and Hull York Medical School, University of York, York; **Dan Mason**, PhD, Bradford Institute for Health Research, Bradford Royal Infirmary, Bradford; **Trevor A. Sheldon**, DSc, Hull York Medical School, University of York, York; **John Wright**, FRCP, Bradford Institute for Health Research, Bradford Royal Infirmary, Bradford, UK

## Abstract

**Background**

There are limited data on detection disparities of common mental disorders in minority ethnic women.

**Aims**

Describe the natural history of common mental disorders in primary care in the maternal period, characterise women with, and explore ethnic disparities in, detected and potentially missed common mental disorders.

**Method**

Secondary analyses of linked birth cohort and primary care data involving 8991 (39.4% White British) women in Bradford. Common mental disorders were characterised through indications in the electronic medical record. Potentially missed common mental disorders were defined as an elevated General Health Questionnaire (GHQ-28) score during pregnancy with no corresponding common mental disorder markers in the medical record.

**Results**

Estimated prevalence of pre-birth common mental disorders was 9.5%, rising to 14.0% 3 years postnatally. Up to half of cases were potentially missed. Compared with White British women, minority ethnic women were twice as likely to have potentially missed common mental disorders and half as likely to have a marker of screening for common mental disorders.

**Conclusions**

Common mental disorder detection disparities exist for minority ethnic women in the maternal period.

Episodes of anxiety and depression (common mental disorders) can recur – following a relapsing and remitting course – or persist becoming chronic and associated with significant disability and limitation of functioning. Mostly, these conditions are managed in primary care, however, general practitioners (GPs) fail to detect up to half of people with depression.^1^ In the UK, minority ethnic groups may have a higher burden of common mental disorders than the majority White population but are less likely to have their disorder detected and treated.^2,3^ Although pregnancy may not increase risk for psychiatric disturbance, common mental disorders during pregnancy can negatively affect the fetus and is the largest risk factor for postnatal depression.^4–6^ For some women, postnatal common mental disorders and subsequent episodes interfere with maternal bonding and affect child development.^7,8^ There are increasing numbers of minority ethnic women in the UK who may be vulnerable in relation to these disorders because of increased risk of poverty, deprivation and physical health problems. This, combined with higher fertility rates in some groups, could mean that a disproportionate number of minority ethnic women have an increased likelihood of undetected common mental disorders before or after pregnancy.

Population prevalence and incidence are most accurately classified using standardised diagnostic interviews establishing presence of diagnoses among affected individuals. Such studies are rare, because they are difficult and expensive to deploy at scale. Although they may include questions about health service use, they are often insufficiently powered to robustly estimate ethnic inequalities with adequate precision.^2^ Larger population surveys using screening instruments validated as capable of identifying groups at elevated risk rarely provide insight into the extent of untreated disorder. More recently researchers have exploited improved access to routine electronically collected health data to calculate population morbidity estimates such as disease prevalence, incidence and risk in healthcare-seeking populations.^9^ This approach benefits from larger samples, but identification and enumeration of undetected or untreated clinical morbidity is only possible in small samples typical of diagnostic validation studies. Detailed sociodemographic information is rarely available. Combining detailed information on mental health and sociodemographics collected for research purposes with routinely collected primary care data is a novel approach that could be used to infer presence of common mental disorders in the primary care setting and examine disparities and risk factors for, and detection of, mental illness. In this study we aimed to use this approach to describe the natural history of common mental disorders as recorded in primary care across the pre-pregnancy, pregnancy and postnatal (maternal) period, describe the characteristics of women with these disorders and those at risk of potentially missed common mental disorders prior to birth, and explore disparities in detection in an ethnically diverse population.

## Method

We analysed data collected from the Born in Bradford (BiB) birth cohort that aims to examine the impact of environmental, psychological and genetic factors on maternal and child health.^10^ Bradford is a northern English city with high levels of socioeconomic deprivation and ethnic diversity. Over 12 000 women were recruited at the Bradford Royal Infirmary at 26–28 weeks pregnancy between 2007 and 2010. Most (83%) filled out a questionnaire reporting sociodemographics, health status and economic situation. Enrolled women consented to linkage of routine data. Ethics approval for the data collection was granted by Bradford Research Ethics Committee (Ref 07/H1302/112).

### Study period and participants

Our study period spanned the 6 months prior to conception, through pregnancy and 1 year after delivery. We also analysed a subsample with at least 3 elapsed years between delivery and February 2013. We distinguish these samples as ‘1-year sample’ and ‘3-year sample’.

**Fig. 1:**
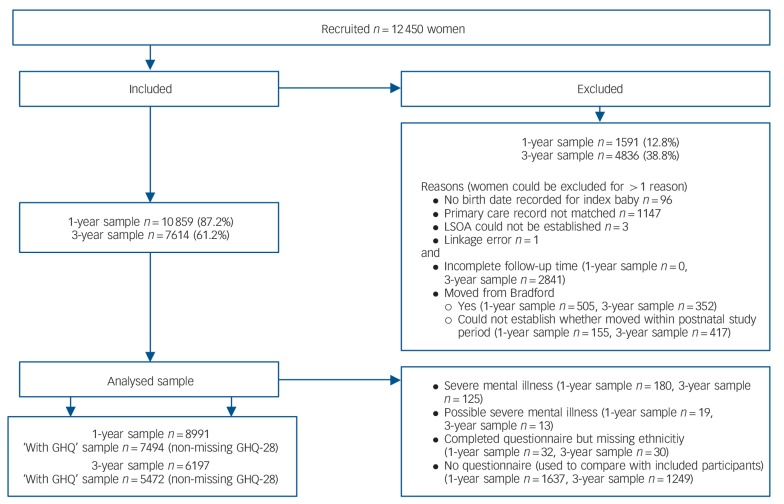
Study flow chart. LSOA, lower super output area (geographical areas of ~1500 households); GHQ, General Health Questionnaire.

We selected one index pregnancy per mother; for women who enrolled multiple pregnancies we selected the index pregnancy as the first enrolled pregnancy with a completed recruitment questionnaire. We used National Health Service (NHS) tracing files to exclude women who relocated from Bradford between enrolment and the end of the study period. This minimised potential unknown missing data bias caused by some women having incomplete GP records because they moved to a practice not using SystmOne (Fig.1). We also excluded women with no linked GP records (9%), any indication of severe mental illness, missing delivery dates, no recruitment questionnaire or no self-reported ethnicity. Just over 27% of women in the 1-year sample were excluded; a further 2841 women were excluded from the 3-year analysis as less than 3 full years had elapsed between the baby's birth and February 2013. Women with a recruitment questionnaire were more likely to be nulliparous, not live in the most deprived areas and have higher healthcare utilisation than those without a recruitment questionnaire (online Table DS1).

### Study data

Data were collected from three sources, the recruitment questionnaire, maternity database and GP records, and these were linked.

#### Recruitment questionnaire

Women were asked to complete the 28-item version of the General Health Questionnaire (GHQ-28).^11^ We adopted the GHQ scoring method, summing the assigned 0-0-1-1 to responses. GHQ data in the BiB cohort have been reported previously.^12^ During the first few months of recruitment, the GHQ-28 was not included in the questionnaire (13% of the 1-year sample). We performed simple imputation (imputed zero) for the 3.3% who did not complete up to four items on the GHQ-28, which, together with women who had a complete GHQ-28, formed a ‘with GHQ’ sample. An ‘impute high’ sensitivity analysis showed a negligible effect of the simple imputation on detection classification.

We classified self-reported ethnicity into three groups: White British, Pakistani and ‘other’. A fourth group comprised women of any ethnicity (mostly Pakistani) who did not complete the recruitment questionnaire in English. These classifications minimised potential language bias.^13^

Covariates originating from the recruitment questionnaire were: age, marital/cohabitation status, country of birth and age at migration, mother's education and employment, the Family Resource Survey Adult Deprivation Questions, the 2010 Index of Multiple Deprivation national rank quintile at recruitment, receipt of means tested benefits and smoking during pregnancy.

#### Maternity database

For women giving birth at the Bradford Royal Infirmary, gestational age at birth and date of birth were obtained from the electronic maternity database and used to calculate date of conception. We performed a simple imputation of gestation as delivery date minus 280 days for the women who did not give birth at the Bradford Royal Infirmary but whose baby's delivery date was obtained from other sources. We used parity recorded in the maternity database as a covariate, backfilling missing data with self-reported parity from the recruitment questionnaire.

#### GP records

Nearly all of Bradford's primary care practices use SystmOne (TPP, Horsforth, Leeds, UK) clinical software in which clinical and administrative terms are classified by Read codes, and prescriptions captured using the British National Formulary dictionary. SystmOne electronic primary care records (‘GP records’) were matched to BiB research records by a third-party data provider using NHS numbers. Matching primary care records were identified for 11 303 (90.8%) BiB research records up to February 2013. We adapted previously published methods to compile lists of Read codes relevant to common mental disorders (signs, symptoms, diagnoses, treatment, referrals, follow-up and screening) and for severe mental illness (psychoses, bipolar disorder, schizophrenia).^14^ We searched participants' GP records for these Read codes, and drugs used to treat common mental disorders, during the study period. We had no access to free-text notes and referral letters because of third-party data protection concerns.

For each month, we used drug prescriptions and Read codes to classify each woman as having markers of detected common mental disorders, having no marker or having markers that we could not classify with any certainty (for example some antipsychotics are also used to treat seizures). Women were thus classified in the following periods: (a) 6 months prior to the date of conception (prenatal); (b) pregnancy (length varied by gestation); (c) prenatal and pregnancy combined (pre-birth); (d) the first postnatal year, and, for the 3-year sample; (e) the second and (f) third postnatal years. We used the combined pre-birth period in most analyses to ensure coverage for prevalent pregnant women managed by midwives not using SystmOne. The very small number of women who only had markers we could not classify (mostly brief prescriptions for antipsychotics, *n* = 8 in 1-year sample) were classified as not having identified common mental disorders during these periods.

We used the presence of a screening code related to common mental disorders in any period as an indication that common mental disorder screening occurred during that period. We used the number of unique days on which a Read code (for anything, not necessarily common mental disorders) had been recorded during the study period as a proxy for healthcare utilisation. We also applied pre-birth common mental disorder screening as a covariate.

#### Classification of women at risk for potentially missed common mental disorders during pregnancy

**Fig. 2:**
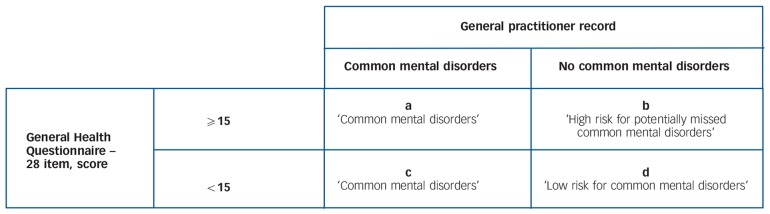
Summary of classification method prior to birth.

For women with no evidence of common mental disorders in their GP record, we used a threshold on the GHQ-28 summary score as an indication of likely psychiatric morbidity. We adopted the threshold ⩾15 since 15 was the 75th centile score for women with GP-identified common mental disorders in the second and third trimesters (Fig. 2). This threshold is high to minimise false positive GHQ-28 screens; the recommended threshold is five to eight.^15,16^ Identified common mental disorders were classified from the GP record only, ignoring the GHQ-28 score.

#### Further pregnancies

Data on further pregnancies in the postnatal period were obtained from (a) subsequent pregnancies enrolled into BiB, and (b) GP records using a Read code search with pregnancy, abortion and fetal death terms, adapting previously published codes.^17^ The sensitivity of method (b) in identifying the (known) index pregnancy was 96.0% (95% CI 95.6–96.4) for the 1-year sample (3-year sample: 97.3%, 95% CI 96.9–97.7). As the exact pregnancy period from conception to delivery could not be accurately determined from GP records, we classed each woman as having, or not having, a pregnancy during the first postnatal year (first 3 years for 3-year sample).

### Statistical analysis

We calculated period prevalence for common mental disorders and screening for common mental disorders as proportions and defined incidence as ‘identified common mental disorders during a period with no prevalence in previous periods’. Incidence rate was calculated per 1000 person-years at risk (PYAR) and we report incidence rate ratios (IRRs) for between-group differences.

We calculated the proportion of women with potentially missed common mental disorders. We weighted for the appropriate positive or negative predictive value (PPV, NPV) of the GHQ-28 to produce more conservative estimates. Values were obtained from a study of English-speaking BiB participants (*n* = 124) evaluated for major and minor depression (prevalence 12.9%: PPV = 0.778, NPV = 0.922, using a cut-off of ⩾15; PPV = 0.393, NPV = 0.948, using ⩾9; details available from the author on request). We used weighted Poisson regression analyses to estimate bivariate differences in risk by ethno-language group.

To avoid residual confounding because of ethnic group differences in socioeconomic status (SES) we stratified multivariate regression analyses by ethno-language group. We estimated unweighted risk models for pre-birth common mental disorders (cells ‘a’ and ‘c’ in Fig. 2) and potentially missed common mental disorders (cell ‘b’) against low-risk women (cell ‘d’) using bivariate and multivariate multinomial logistic regression. We conducted similar comparisons between detected (cells ‘a’ and ‘c’) and potentially missed common mental disorders (cell ‘b’) using logistic regression. We used augmented chained equations (M = 10) as implemented in Stata's ‘mi’ suite of commands to generate and estimate stratified multiply imputed data-sets to account for missing questionnaire data. We assumed data were either missing completely at random (for example GHQ-28 missing by design in the first few enrolment months) or missing at random with missingness associated with observed covariates. We imputed missing GHQ-28 categorised scores, classification of pre-birth risk and covariate data using outcome, covariate and design variables in the imputation model. We report relative risk ratios (RRRs, bivariate RRRs and adjusted RRRs) selecting variables with a bivariate association *P*⩽0.1 for adjusted models. Results using the ‘with GHQ’ sample, both weighted and unweighted, were similar, although point estimates were generally less conservative, compared with imputed results. To avoid overestimating associations between dependent variables and covariates and to make use of all possible information we report main results using the imputed data-sets only. Lastly, we calculated the within-group prevalence of postnatal common mental disorders by pre-birth risk status.

We ran two sensitivity analyses on the threshold used to classify women at risk for potentially missed common mental disorders: (a) using ⩾9 on the GHQ-28 (median score of women with identified common mental disorders in the second and third trimester), and (b) using the highest 10% of GHQ-28 scores within each ethno-language group. We used these to check for potential bias caused by (a) women with very high GHQ-28 scores having different characteristics from those with moderate or high GHQ scores, with no change in common mental disorder risk, and (b) variation in scores between ethno-language groups because of measurement error. We present 95% confidence intervals around prevalence, incidence and risk estimates and used Stata release 12 to conduct all analyses.

## Results

### Description of the study population

Our analytic sample comprised 8991 women (6197 for the 3-year sample), 72.2% (49.8% for the 3-year sample) of recruited women (Fig. 1). Table 1 shows the demographic and health behaviour characteristics and Table 2 the socioeconomic characteristics of all participants (see online Table DS2 for a version that includes a wider range of characteristics). Pakistani women had higher GHQ-28 scores and higher healthcare utilisation. There was variation in parity and further pregnancy between groups (Table 1) but most women lived in deprived areas (Table 2). Characteristics of the 3-year sample were similar to the 1-year sample (online Table DS3).

**Table 1:** Demographic and health behaviour characteristics of participants included for 1-year follow-up^a^

	Ethnicity (language of enrolment)				
	White British (English) (*n* = 3546)	Pakistani (English) (*n* = 2602)	Other (English) (*n* = 1209)	Any ethnicity (Not English) (*n* = 1634)	Total (*n* = 8991)
Age, years: mean (s.d.)	26.7 (6.1)	27.6 (5.1)	28.0 (5.5)	27.8 (5.4)	27.3 (5.6)

Parity, *n* (%)					
Nulliparous	1754 (50.6)	937 (37.2)	597 (50.2)	517 (32.3)	3805 (43.4)
1	1024 (29.5)	613 (24.3)	329 (27.7)	401 (25.1)	2367 (27.0)
2–3	602 (17.4)	794 (31.5)	237 (19.9)	518 (32.4)	2151 (24.5)
4+	87 (2.5)	176 (7.0)	27 (2.3)	163 (10.2)	453 (5.2)
Missing	79 (2.2)	82 (3.2)	19 (1.6)	35 (2.1)	215 (2.4)

Marital/cohabitation status, *n* (%)					
Married	1100 (31.1)	2370 (91.2)	860 (71.1)	1563 (96.0)	5893 (65.7)
Cohabiting	1401 (39.6)	15 (0.6)	163 (13.5)	4 (0.3)	1583 (17.6)
Not living with a partner	1037 (29.3)	213 (8.2)	186 (15.4)	62 (3.8)	1498 (16.7)
Missing	8 (0.2)	4 (0.2)	0	5 (0.3)	17 (0.2)

Country of birth and age at migration, *n* (%)					
Born in UK	3486 (98.5)	1734 (67.4)	476 (40.1)	32 (2.0)	5728 (64.3)
Immigrated before age 16	40 (1.1)	394 (15.3)	134 (11.3)	73 (4.6)	641 (7.2)
Immigrated 16 or older	14 (0.4)	433 (16.7)	578 (48.7)	1501 (93.5)	2536 (28.5)
Missing	6 (0.2)	31 (1.2)	21 (1.7)	28 (1.7)	89 (1.0)

Further pregnancies in the 12-month postnatal period, *n* (%)					
No	3299 (93.0)	2277 (87.5)	1126 (93.1)	1375 (84.2)	8077 (89.8)
Yes	247 (7.0)	325 (12.5)	83 (6.9)	259 (15.9)	914 (10.2)

Smoking during pregnancy, *n* (%)					
No	2329 (65.7)	2461 (94.8)	1067 (88.4)	1621 (99.4)	7478 (83.3)
Yes	1214 (34.3)	134 (5.2)	140 (11.6)	10 (0.6)	1498 (16.7)
Missing	3 (0.1)	7 (0.3)	2 (0.2)	3 (0.2)	15 (0.2)

General practitioner visits,^b^ mean (s.d.)	24.3 (13.0)	27.5 (15.1)	22.6 (12.0)	25.4 (13.6)	25.2 (13.7)

a. See online Table DS2 for a version of this table that includes a wider range of characteristics. Proportions presented in categories are the proportion of observed (non-missing) data, missing is the proportion of data missing overall.

b. Proxy used for the number of visits between 6 months prior to conception and 1 year post-delivery.

**Table 2:** Socioeconomic characteristics of participants included for 1-year follow-up^a^

	n (%)				
	Ethnicity (language of enrolment)				
	White British (English) (*n* = 3546)	Pakistani (English) (*n* = 2602)	Other (English) (*n* = 1209)	Any ethnicity (Not English) (*n* = 1634)	Total (*n* = 8991)
Mother's education^b^					
⩾A-level equivalent	644 (18.2)	711 (27.4)	484 (40.3)	361 (22.2)	2200 (24.5)
A-level equivalent	614 (17.3)	498 (19.2)	172 (14.3)	36 (2.2)	1320 (14.7)
5 GCSE equivalent	1229 (34.7)	832 (32.0)	256 (21.3)	483 (29.7)	2800 (31.1)
<5 GCSE equivalent	699 (19.7)	406 (15.6)	135 (11.2)	706 (43.4)	1946 (21.7)
Other education qualification	319 (9.0)	124 (4.8)	138 (11.5)	17 (1.0)	598 (6.7)
Unknown qualification	37 (1.0)	27 (1.0)	20 (1.4)	24 (1.5)	105 (1.2)
Missing	4 (0.1)	4 (0.2)	7 (0.6)	7 (0.4)	22 (0.2)

Mother's employment status					
Currently employed	2321 (65.5)	922 (35.5)	713 (59.1)	81 (5.0)	4037 (45.0)
Previously employed	933 (26.3)	1010 (38.9)	307 (25.5)	258 (15.8)	2508 (27.9)
Never employed	290 (8.2)	665 (25.6)	186 (15.4)	1292 (79.2)	2433 (27.1)
Missing	2 (0.1)	5 (0.2)	3 (0.3)	3 (0.2)	13 (0.1)

Index of Multiple deprivation quintile compared with national rank (2010) for pregnancy address						
Most deprived	1806 (50.9)	1976 (75.9)	810 (67.0)	1381 (84.5)	5975 (66.5)
2	766 (21.6)	419 (16.1)	246 (20.4)	185 (11.3)	1616 (18.0)
3	637 (18.0)	182 (7.0)	122 (10.1)	62 (3.8)	1003 (11.2)
4	217 (6.1)	15 (0.6)	18 (1.5)	5 (0.3)	255 (2.8)
Least deprived	120 (3.4)	10 (0.4)	13 (1.1)	1 (0.1)	144 (1.6)

In receipt of means tested benefits					
No	2227 (63.0)	1366 (52.6)	851 (70.5)	927 (56.9)	5371 (59.9)
Yes	1306 (37.0)	1230 (47.4)	356 (29.5)	701 (43.1)	3593 (40.1)
Missing	13 (0.4)	6 (0.2)	2 (0.2)	6 (0.4)	27 (0.3)

a. See online Table DS2 for a version of this table that includes a wider range of characteristics. Proportions presented in categories are the proportion of observed (non-missing) data, missing is the proportion of data missing overall.

b. GCSEs are qualifications obtained at the end of compulsory education at age 16, A-levels are qualifications achieved after successful completion of a further 2 years' full-time school after compulsory education ends at age 16.

### Identified pre-birth common mental disorders

#### Prevalence

**Fig. 3:**
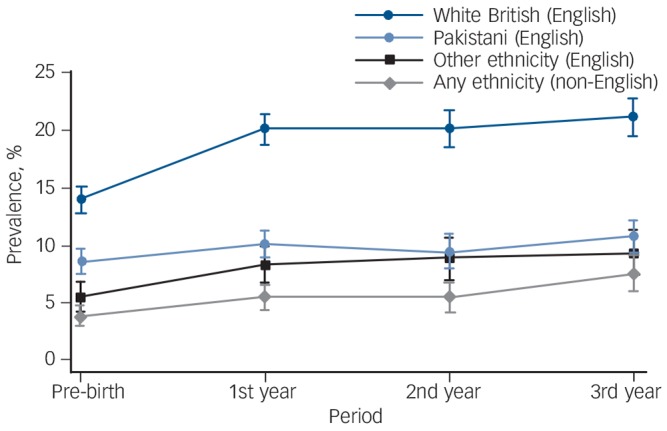
Period prevalence of identified common mental disorders. Pre-birth and first year *n* = 8991; second and third year *n* = 6197.

Overall, 9.5% (95% CI 8.9–10.1) had a marker of common mental disorders on their pre-birth record. Prevalence in the first postnatal year was 13.1% (95% CI 12.4–13.7), second year 12.8% (95% CI 11.9–13.6) and third 14.0% (95% CI 13.2–14.9). Women with further pregnancies in the study period were more likely to have identified common mental disorders (for example first year 17.3% *v*. 12.6%). White British women had around double the prevalence of common mental disorders compared with minority ethnic women at each period (Fig. 3). Prevalence appeared stable throughout enrolment.

#### Incidence

Incident identified common mental disorders during pregnancy was 37.5 per 1000 PYAR (95% CI 33.1–42.5), in the first postnatal year 102.4 (95% CI 95.5–109.8), second year 64.9 (95% CI 58.1–72.4) and third 68.2 (95% CI 61.1–76.2). White British women had around double the incidence at each period, for example IRR = 1.95 (95% CI 1.64–2.32) compared with Pakistani women for the first postnatal year, and 3.33 (95% CI 2.60–4.31) compared with women not using English (online Table DS4).

### Screening for common mental disorders

Although fewer than 13% of women had Read codes in their records indicating screening and case-finding for common mental disorders in the first postnatal year, twice as many White British women had these codes compared with minority ethnic women.

### Women at risk for potentially missed pre-birth common mental disorders

Pakistani women were more likely to have GHQ-28 scores ⩾15 than White British women (Table 3). However, compared with White British women, minority ethnic women had fewer high-scorers with identified common mental disorders and, therefore, 2.3 to 2.7 times the risk of potentially missed common mental disorders. Attenuated, but still statistically significant (*P*<0.001), disparities were observed after varying the threshold to ⩾9 (RRR of potentially missed common mental disorders between 1.80 and 2.10) and to the 90th centile (RRR = 1.38–1.94).

**Table 3:** Common mental disorders and General Health Questionnaire – 28 item (GHQ-28) scores^a^

	Ethnicity (language of enrolment), *n* (%)				Comparison, relative risk ratio (95% CI)		
	White British (English)	Pakistani (English)	Other (English)	Any ethnicity (non-English)	Pakistani *v*. White British	Other *v*. White British	non-English *v*. White British
GHQ-28 score							
<15	2850 (94.2)	1940 (87.9)	947 (92.6)	1182 (95.5)	1	1	1
⩾15	177 (5.9)	266 (12.1)	76 (7.4)	56 (4.5)	**2.06 (1.71–2.49)**	1.27 (0.97–1.66)	0.77 (0.57–1.04)

GHQ-28 score ⩾l5^b,c^							
No common mental disorders	112 (57.3)	209 (74.0)	65 (82.1)	45 (76.1)	1	1	1
Common mental disorders	65 (42.7)	57 (26.0)	11 (17.9)	11 (23.9)	**0.61 (0.46–0.80)**	**0.42 (0.24–0.73)**	**0.56 (0.33–0.96)**

Common mental disorder status^b,c^							
Identified	424 (83.0)	196 (54.7)	64 (55.9)	53 (60.2)	1	1	1
Potentially missed	112 (17.1)	209 (45.3)	65 (44.1)	45 (39.8)	**2.66 (2.17–3.26)**	**2.59 (2.00–3.35)**	**2.33 (1.74–3.14)**

a. ‘With GHQ’ sample, poisson regression. Results in bold are significant.

b. *n* are unweighted at base.

c. Proportions and regression weighted for predictive values of the GHQ-28 at ⩾15 for major and minor depression.

Overall, we estimated between 31.3% (95% CI 28.7–33.8; weighted for the threshold ⩾15) and 46.8% (95% CI 44.7–49.0; weighted for the threshold ⩾9) of individuals with pre-birth common mental disorders were potentially missed.

### Factors associated with common mental disorders status

Tables of estimates are provided in online Tables DS5–8.

#### Women with identified pre-birth common mental disorders

Compared with women at lower risk of common mental disorders, women with detected common mental disorders were less likely to be married, more likely to have lower SES, increased healthcare utilisation and have pre-birth common mental disorders screening. Confidence intervals were wide around screening estimates, reflecting the very low levels of recorded screening.

#### Women at risk for potentially missed pre-birth common mental disorders

Compared with women at lower risk, across all ethnic groups, those with potentially missed common mental disorders had lower SES. For White British women, increased healthcare utilisation was associated with potentially missed common mental disorders, with a similar non-statistically significant trend for Pakistani women. For the women who did not use English the potentially missed common mental disorders group were statistically more likely to have screening than the low-risk group, but fewer than 1% of non-English-speaking women received any screening, and in the other ethnic groups women with potentially missed common mental disorders seemed to be as likely as the lower risk of common mental disorders group to have had pre-birth screening. On changing the threshold to ⩾9, increased healthcare utilisation was associated with potentially missed common mental disorders for Pakistani women (adjusted RRR = 1.78, 95% CI 1.39–2.26).

Compared with women with identified common mental disorders, women with potentially missed common mental disorders who completed English questionnaires were less likely to have been screened, and, with the exception of White British women, were less likely to have high levels of healthcare utilisation. There was little consistent evidence across the ethnic groups of lower or higher SES in women with potentially missed common mental disorders compared with those with detected common mental disorders. White British women with potentially missed common mental disorders were more likely to be younger and cohabitating than those identified. There was no statistically significant variation in any risk factor, including screening, between those potentially missed and those identified in women who did not use English, except after changing the threshold to ⩾9, screening was less likely in those with potentially missed common mental disorders (adjusted RRR = 0.14, 95% CI 0.04–0.57). All these results were robust to sensitivity analyses.

### Postnatal common mental disorders

**Fig. 4:**
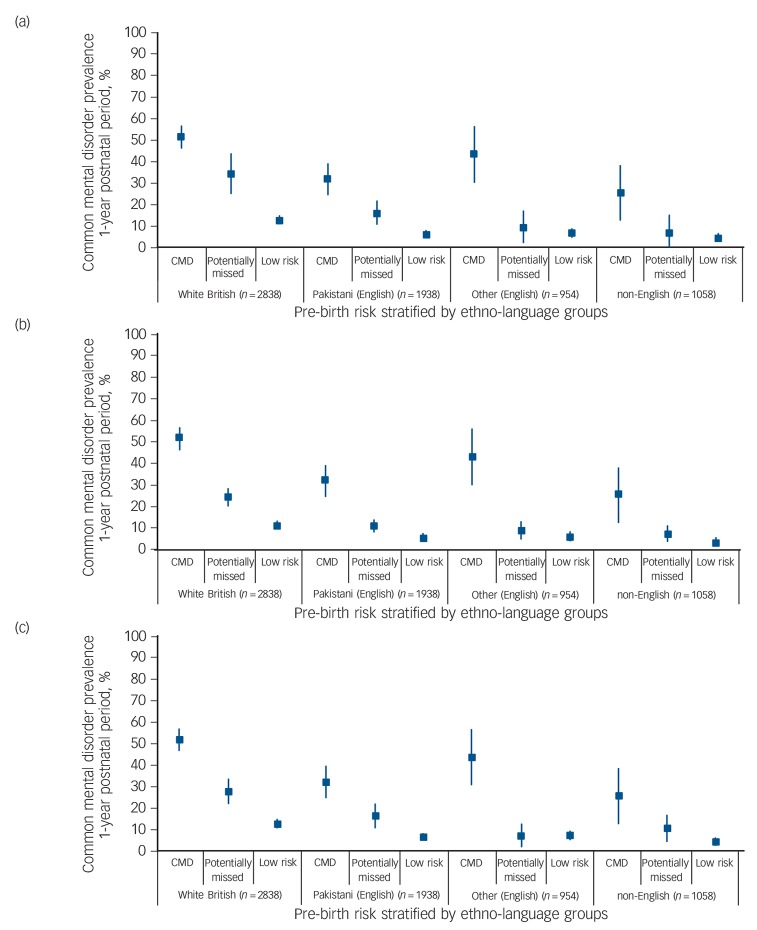
Prevalence of 1-year postnatal common mental disorders by pre-birth risk status. (a) Risk classified at General Health Questionnaire – 28 item (GHQ-28) threshold ⩾15; (b) risk classified at GHQ-28 threshold ⩾9; (c) risk classified at within-group GHQ-28 90th centile. ‘With GHQ’ sample, women without further pregnancies in 1-year postnatal period. CMD, common mental disorders.

Prevalence of postnatal common mental disorders was highest in the women with detected pre-birth common mental disorders (Fig. 4(a)). White British and Pakistani women with potentially missed pre-birth common mental disorders also had an elevated prevalence of postnatal common mental disorders compared with low-risk women. This pattern of increased detection was not evident for women in the other two ethno-language groups. Varying the threshold attenuated but did not alter results (Fig. 4(b) and (c)).

## Discussion

### Main findings

Using Read codes and prescriptions in the electronic GP record, the estimated prevalence of common mental disorders for the pre-birth period was 9.5% (13.1% in the first postnatal year, 12.8% in the second, and 14.0% in the third). White British women had twice the prevalence and incidence of psychiatric morbidity compared with minority ethnic women. Based on a self-reported screening scale with scores measuring distress at levels likely to indicate caseness our estimate of the overall proportion of missed cases of pre-birth common mental disorders was between 31.3% (95% CI 28.7–33.8) and 46.8% (95% CI 44.7–49.0). Minority ethnic women had twice the rate of potentially missed cases and half the volume of screening records. Across all ethnic groups, lower SES was associated with pre-birth morbidity, whether detected or not. Detection of this morbidity was associated with screening, at least for women using English, although low levels of screening preclude firm conclusions. Increased healthcare utilisation was more likely in women with detected common mental disorders compared with women with low risk. The relationship between healthcare utilisation for the women who were potentially missed compared with detected and low risk varied by ethnic group. Ethnic disparities in detection of significant clinical morbidity continued postnatally.

### Strengths and limitations

BiB is a large cohort study thus rates could be estimated with some precision with results robust to a variety of methodological assumptions. Linkage between routine data and BiB avoids traditional attrition problems and we limited missing routine data by excluding women who moved. To minimise bias caused by variation in recording by GP, or time, we broadly defined potential disorders.^18^ We consider it unlikely that more minority ethnic women have their mental health managed by health visitors and midwives outside the electronic record, and, regardless, GPs should be notified of suspected cases.^19^ Our study has some limitations. The quantity of missing data from the primary care data-set is unknown. Morbidity or treatment noted by free-text or letter were not available, meaning that results could have been related to differences in free-text recording by GPs caring for either minority ethnic or majority women. An incomplete history of common mental disorders in the medical record and a lack of causal ordering meant we could not be certain which screening records were case-finding for the incident disorder and which were used for monitoring existing psychopathology. We acknowledge the limitations of the GHQ-28 only being administered once and could not find a report of its case-finding properties during pregnancy that would enable us to apply weights for anxiety and mixed episodes. NHS guidance documents advise screening maternal populations for depression,^19,20^ but only for anxiety after our study ended,^20^ hence ethnic differences in specific disorder prevalence may have affected our results. We did not aim to identify all women with potentially missed common mental disorders and our predictions contain unknown error quantities, although we presented weighted prevalence and believe we have under- rather than overestimated ethnic differences. The regression analyses may overstate relationships between covariates and these disorders because of multiple testing. It is difficult to quantify the impact of excluded cases because of missing ethnicity along with missing GHQ data. Pooling smaller ethnic groups may have masked important within-group differences in risk.^12^ Our proxy marker for GP contact could be unreliable because of varying quantities of administrative codes. Finally, Bradford is a disadvantaged city with low socioeconomic diversity and it is unclear how findings might translate to more economically diverse areas, or general populations.

### Comparison with findings from other studies

We adopted a general approach to morbidity (common mental disorders) in contrast to most research that focuses on either depression or anxiety because mixed episodes are common,^21^ single episodes are less reliably separated in the GP record using our coding criteria and sequelae for children are equally high.^8^ We included descriptive terms and studied an economically disadvantaged community, a risk factor for common mental disorders. Thus, in the absence of underdetection, prevalence and incidence in our study should be comparatively high. However, our period prevalence estimates are low compared with studies in a review of maternal depression (not including anxiety) assessed using diagnostic criteria.^6^ They are also low compared with a BiB subsample assessed in a depression diagnostic accuracy study,^22^ implicating common mental disorder under-recognition within the primary care setting. Studies using GP records typically report fewer cases than diagnostic studies because of underdetection and coding problems.^18,23^ Prevalence, however, in our study, still appears low compared with analyses of a primary care research data-set of child-bearing age women who had Read-coded anxiety diagnoses or symptoms,^9^ or depression diagnoses.^24^ Financial incentives available to GP practices for contributing to primary care research data-sets might be associated with more consistent coding, or possibly better rates of case-finding, than in the practices contributing to our study. Populations represented by practices contributing to primary care research data-sets may also be different to BiB (on average they will serve more advantaged populations), pathology in maternal populations may be different to non-maternal women or there may be pervasive coding differences during pregnancy across GP data. Incidence in our study is high compared with analyses of child-bearing age women in a primary care research data-set using Read codes to identify anxiety diagnoses or symptoms,^9,18^ or depression diagnoses.^24^ Although this could indicate the absence of a problem of absolute underdetection in our study, it could also be caused by underrecording of pregnancy prevalence, for example by midwives outside the GP record, which would falsely inflate future incidence. However, incidence in our study is low or comparable with an analysis of depression diagnoses and depression-related prescriptions in the first postnatal year.^25^ This may reflect a genuinely higher risk of incident disorder in maternal populations, similar problems in both studies in accurately accounting for pre-birth prevalence, or the effect of including prescriptions to aid in case-finding. Differences in study populations and analytic and case-finding methodologies make comparisons about our study's findings and others' very challenging. Despite these comparative differences, our estimate of missed diagnoses does agree with other non-maternal and postnatal studies.^1,26^ Although the evidence base is limited,^27^ there is little to suggest that Pakistani women have a lower common mental disorder burden,^3,28–30^ thus, although cultural differences in responses to screening questionnaires may account for some variation, we consider our results to indicate health disparity rather than a genuine difference in prevalence.

### Implications

We noted extremely low levels of screening overall, presumably because of non-electronic recording by midwives or health visitors. Our results, however, indicate that case-finding in disadvantaged women has the potential to be successful, and, if systematically and rigorously employed, might reduce detection disparities. The lower observed levels of screening for ethnic minorities could be related to the lack of culturally validated and non-English screening instruments,^31^ inhibiting case-finding activity during consultations. There may also be cultural sensitivities in asking about psychological symptoms, and patient reporting of symptoms, where there is potential stigma associated with such problems. Screening, case-finding and improved detection in itself does not improve mental health and needs to be accompanied by effective intervention.^32^

White British women who were potentially missed cases had greater healthcare utilisation than those with low common mental disorder risk, with borderline statistical significance for English-speaking Pakistani women. This possibly indicates higher levels of physical health problems and minor psychiatric diagnoses,^33^ or increased visits for the same state of health. Indeed, across ethnic groups the level of healthcare utilisation was similar between potentially missed and detected cases. Ethnic minorities access primary care services at similar rates to majority populations, but may be less likely to consult GPs with a mental health concern, indicating that consultations are less effective for mental healthcare-seeking.^2,30,34,35^ We found that non-English language users were least likely to be identified with mental health problems. The effect of language in consultations will vary by practice and GP, but differences could be minimised by improved access to translators. Language could also be a proxy for more recent immigrants, reported to have better mental health,^36^ *prima facie* observed as decreased prevalence in our study, but a reduced risk for mental disorder should not be assumed in any consultation. Disorder identification for any ethnic group is likely to be influenced by both patient and professional barriers,^37^ and GPs working in deprived inner cities need to beware normalisation of distress because of a high volume of pervasive ‘misery’.^38,39^ Recent and established immigrant populations may experience and express psychopathology somewhat differently, and variation in clinicians' cultural competency may result in health inequality.^29,40,41^

### Directions for future research

Our study has highlighted several areas for future research. Prospective longitudinal research is needed to understand how and why some women ‘fall through the gaps’ of continued care. The effects of potentially missed common mental disorders on children's outcomes need to be quantified. Studies into the content of health professional encounters with minority/majority and disadvantaged/advantaged women at risk for psychiatric disorder are needed, with a view to improving the quality of case-finding in routine visits and reducing disparities. Similar investigations into how screening is recorded would help unpack our unanswered question about whether the low levels of screening observed in this study are a result of limited screening, screening undertaken by other health professionals or variation in recording. This would provide evidence to feed into the assessment of robustness and generalisability of effectiveness and cost-effectiveness estimates for screening programmes. Culturally specific and cross-cultural screening instruments need validation, and interventions in their use and effect should be evaluated. More definitive research is required to clarify whether, in vulnerable maternal populations, less healthcare is sought or healthcare-seeking is less effective. Generally, causal studies into factors that predict identification and reduce inequality are required. Finally, routine clinical information can be a rich source of research data but our study highlights the potential for underreporting and masking of substantial health inequalities; greater understanding of potential bias in routine data sources is needed.
